# Retraction Note: CircHIPK3 promotes colorectal cancer growth and metastasis by sponging miR-7

**DOI:** 10.1038/s41419-024-06761-z

**Published:** 2024-05-29

**Authors:** Kaixuan Zeng, Xiaoxiang Chen, Mu Xu, Xiangxiang Liu, Xiuxiu Hu, Tao Xu, Huiling Sun, Yuqin Pan, Bangshun He, Shukui Wang

**Affiliations:** 1 General Clinical Research Center, Nanjing First Hospital, Nanjing Medical University, 210006 Nanjing, China; 2 School of Medicine, Southeast University, 210009 Nanjing, China

Retraction to: *Cell Death and Disease* 10.1038/s41419-018-0454-8, published online 16 March 2018

The Editor-in-Chief has retracted this article after concerns were raised about some of the data reported. Specifically:

- There appears to be an area of overlap between the si-circHIPK3#1 panel of HCT116 in Fig. 3F and the HT29 panel of miR-NC in Fig. 5D.
- In Fig. 5B, there appears to be an area of overlap between the miR-7+circHIPK3 panel of HCT116 and the miR-NC+circHIPK3 panel of HT29.
- There appears to also be significant similarities between the two panels mentioned above and the top-left panel of Fig. 3D.
- In Fig. 5G, there is evidence of a potential splice in channel YY1 of HCT116.

The Editor-in-Chief no longer has confidence in the article’s findings and conclusions.

Kaixuan Zeng disagrees with this retraction. The remaining authors did not respond to correspondence from the publisher about this retraction.

